# Comparison of Dye Spread Pattern and Nerve Involvement between Suprainguinal and Infrainguinal Fascia Iliaca Blocks with Different Injectate Volumes: A Cadaveric Evaluation

**DOI:** 10.3390/medicina60091391

**Published:** 2024-08-25

**Authors:** Tae-Hyeon Cho, Byongnam Jun, Hun-Mu Yang, Shin Hyung Kim

**Affiliations:** 1 Department of Anatomy, College of Korean Medicine, Semyung University, Jecheon-si 27136, Republic of Korea; gods176@semyung.ac.kr; 2 Translational Research Unit for Anatomy and Analgesia, Seoul 03722, Republic of Korea; yanghm@yuhs.ac; 3 Department of Anesthesiology and Pain Medicine, Yonsei University College of Medicine, Seoul 03722, Republic of Korea; bnjun@yuhs.ac; 4 Translational Laboratory for Clinical Anatomy, Department of Anatomy, Yonsei University College of Medicine, Seoul 03722, Republic of Korea; 5 Surgical Anatomy Education Center, Yonsei University College of Medicine, Seoul 03722, Republic of Korea

**Keywords:** cadaveric evaluation, fascia iliaca compartment block, injectate volume, regional anesthesia, ultrasonography

## Abstract

*Background and Objectives*: Fascia iliaca compartment block (FICB) is an effective and relatively safe technique in perioperative pain management for hip surgery. However, blockade of the obturator nerve (ON) using this technique remains controversial. This study aimed to compare dye spread patterns and nerve involvement in the suprainguinal FICB (S-FICB) and infrainguinal FICB (I-FICB) approaches using different volumes of dye. *Materials and Methods*: Following randomization, 6 S-FICBs and 6 I-FICBs were performed on the left or right sides of 6 unembalmed cadavers. For each block, 30 mL or 60 mL of dye solution was injected. The extent of dye spreading and the staining pattern in the lumbar plexus branches were investigated using anatomical dissection. *Results*: Twelve injections were successfully completed. The lateral femoral cutaneous nerve (LFCN) and femoral nerve (FN) were consistently stained in all injections. Extended dye spread toward lumbar plexus branches was observed volume-dependently in S-FICBs. However, I-FICBs with an increased volume only showed dye spreading in the caudad direction limited to within the fascia iliaca. When 30 mL of dye was used, the ON was not stained with either approach. A stained ON was only observed in S-FICBs when 60 mL of dye was used. *Conclusions*: In this cadaveric evaluation, the ON was not stained in either FICB approach with the volume of injectate commonly used in clinical practice. The S-FICBs but not I-FICBs using a high volume of injectate resulted in extended spreading to the lumbar plexus branches.

## 1. Introduction

Lumbar plexus block, directly targeting the psoas compartment using the posterior approach, has been used as a method of analgesia and regional anesthesia for decades in patients undergoing hip surgery; however, the use has not been routine, mainly due to lack of experience and unidentified complications [1]. The posterior lumbar plexus block has been associated with several serious complications such as epidural spread or spinal anesthesia, intraperitoneal injection, and retroperitoneal hematoma [2]. Patients receiving anticoagulation therapy or diagnosed with coagulopathy may not be suitable candidates for a posterior lumbar plexus block. By contrast, an anterior approach, such as the fascia iliaca compartment block (FICB), has gained popularity due to high success and low block-related complications [3]. Therefore, FICB is often regarded as a safe and relatively simple alternative to conventional femoral nerve (FN) block and lumbar plexus block [4,5,6].

The FICB anesthetizes the three branches of the lumbar plexus simultaneously: the FN, lateral femoral cutaneous nerve (LFCN), and obturator nerve (ON) [3,4]. The two approaches of suprainguinal FICB (S-FICB) and infrainguinal FICB (I-FICB) have been introduced in clinical practice [4]. Ultrasound-guided S-FICB was introduced after I-FICB as a conventional approach, but both remain controversial regarding coverage of the ON [6,7,8]. We hypothesized that a higher volume of injectate than conventionally administered in FICB would allow more extensive nerve involvement from the lumbar plexus. Specifically, the volume-dependent injectate spreading patterns and ON involvement have not been compared between the two FICB approaches in cadavers.

Therefore, in this cadaveric study, the dye spread patterns and involvement of lumbar plexus branches including the ON in S-FICB and I-FICB were compared using a commonly used volume (30 mL) or high volume (60 mL) of dye.

## 2. Materials and Methods

S-FICB or I-FICB were performed on 6 unembalmed fresh-frozen cadavers (3 males and 3 females; age: 71–89 years; mean age: 80.2 years). The Institutional Review Board approved the study for exemption from formal review (No. 4-2019-0237). The scientific staff at the Surgical Anatomy Education Center at Yonsei University College of Medicine (YSAEC) independently chose the cadavers. All cadavers used in the present study were legally donated to the YSAEC and had experienced no trauma, operative procedure, or pathologic condition in the pelvic and femoral region. Each cadaver received one I-FICB and one S-FICB, with the body side and dye volume of 30 mL or 60 mL decided randomly according to a computer-generated random number list.

### 2.1. S-FICB Procedure

**Figure 1:**
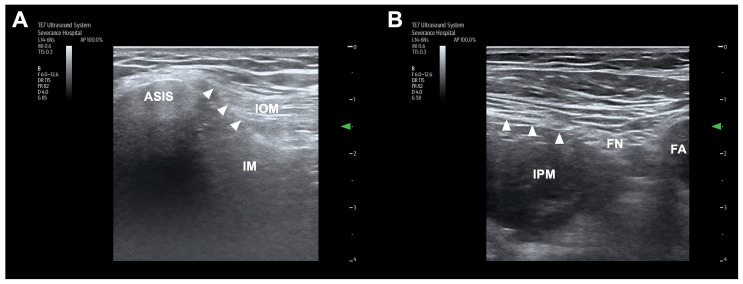
Ultrasound anatomy for the suprainguinal (**A**) and infrainguinal (**B**) fascia iliaca block. Arrowheads indicate the fascia iliaca. ASIS, anterior superior iliac spine; IM, iliacus muscle; IOM, internal abdominal oblique muscle; IPM, iliopsoas muscle; FN, femoral nerve; FA, femoral artery.

All cadavers were placed in the supine position, and injections were performed by a single experienced anesthesiologist specializing in regional anesthesia and pain medicine. A TE7 ultrasound unit (Mindray Bio-Medical Electronics, Shenzhen, China) with a high-frequency linear probe (4–16 MHz) was used. The ultrasound-guided S-FICB procedure followed the standard protocol (Figure 1A) [9]. The ultrasound probe was positioned longitudinally at the level of the anterior superior iliac spine and was moved medially to identify the fascia iliaca and sartorius, iliopsoas, and internal oblique muscles. After identifying the ‘bowtie sign’ formed by the muscle fasciae, an 80 mm 22-gauge needle was introduced 1 cm cephalad to the inguinal ligament. Using an in-plane approach, the needle tip was positioned beneath the fascia iliaca, and hydrodissection was used to separate the fascia iliaca from the iliacus muscle. The needle was further advanced in this space in a cranial and slightly dorsal direction. Correct needle placement was confirmed on the basis of the separation between the fascia iliaca and the iliac muscle and the presence of cranial spread of injectate beneath the fascia iliaca. After confirming the correct localization of the needle tip, 30 mL or 60 mL of dye solution was injected during a standardized period of 2 min or 4 min, respectively. The dye solution was either a 30.0 mL mixture of 27 mL distilled water, 2 mL latex solution (Duksan, Ansan, Republic of Korea) and 1 mL green ink (Polysciences, Inc., Warrington, PA, U.S.), or a 60 mL mixture of 54 mL distilled water, 4 mL latex solution, and 2 mL green ink.

### 2.2. I-FICB Procedure

The ultrasound-guided I-FICB was performed following the standard protocol (Figure 1B) [10]. The ultrasound probe was positioned below the inguinal ligament within the inguinal crease and oriented transversely. Several ultrasound landmarks were identified, including the iliac muscle, sartorius muscle, fascia iliaca, FN, and femoral artery. Subsequently, the probe was moved laterally along the fascia iliaca toward its junction with the medial border of the sartorius muscle. An 80 mm 22-gauge needle was advanced in-plane, beginning from the lateral side and progressing medially, finally piercing the fascia iliaca at the point where it intersects with the iliac muscle and the medial border of the sartorius muscle within the inguinal crease. Correct needle placement was confirmed on the basis of the separation of the fascia iliaca from the iliopsoas muscle with the injectate spreading toward the FN medially and the iliac crest laterally. After confirming the correct localization of the needle tip, 30 mL or 60 mL of dye solution was injected during a standardized period of 2 min or 4 min, respectively.

### 2.3. Anatomical Dissection Procedure

Two experienced anatomists, each with over 10 years of experience in gross anatomy (T.-H.C. and H.-M.Y.), were blinded to the volume of injectate used in each approach. The dissection protocol was followed in all cadavers, and dissection started no later than 1 h after injection. Bilateral dissections were concurrently performed to eliminate the possibility of excess dye spreading on one side due to varying dissection onset times. The skin, subcutaneous tissue, deep fascia, and superficial vessels were cautiously removed. Next, the abdomen, inguinal region, femoral region, and its related structures including nerves, arteries, and veins, were exposed. In the abdomen and inguinal region, the fascia iliaca over the iliopsoas muscle was dissected on the side injected with dye. The lumbar plexus branches (iliohypogastric nerve, ilioinguinal nerve, genitofemoral nerve (GFN), LFCN, FN, and ON) overlying the anterior aspect of the iliopsoas muscle and the anterior and posterior abdominal wall muscle were identified. To observe the ON and root of the lumbar plexus, the psoas major muscle was retracted laterally. In the femoral region, the femoral triangle and its related structures were exposed. The LFCN, FN, and anterior and posterior branches of the ON were identified. Positive cases were those with nerves deeply stained with dye as determined by both anatomists and one anesthesiologist.

## 3. Results

A total of 12 FICBs, including six S-FICBs (three injections using 30 mL of dye and three injections using 60 mL of dye) and six I-FICBs (three injections using 30 mL of dye and three injections using 60 mL of dye), were successfully performed.

### 3.1. Dye Spreading Patterns in the S-FICBs

**Figure 2:**
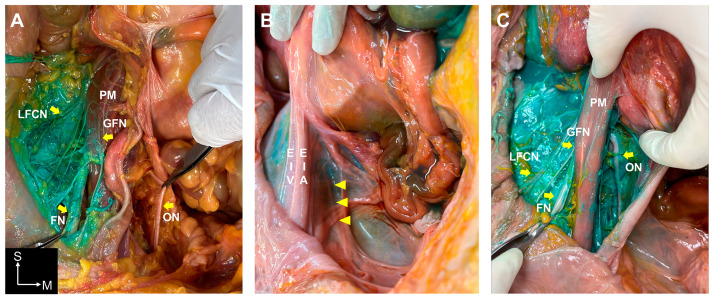
Comparison of the dye spreading patterns in the suprainguinal fascia iliaca compartment block (S-FICB) using 30 mL and 60 mL of dye solution. (**A**). In the S-FICB using 30 mL of dye, the genitofemoral nerve (GFN), lateral femoral cutaneous nerve (LFCN), and femoral nerve (FN) were stained, but the obturator nerve (ON) was not. The FN (left) and ON (right) are held with forceps, respectively. (**B**). In the S-FICB using 60 mL of dye, dye spread was observed beneath the external iliac artery and vein (EIA and EIV). Yellow arrowheads indicate the ON covered by the pelvic fascia. (**C**). In the S-FICB using 60 mL of dye, ON staining was confirmed in the retroperitoneal space, specifically on the posteromedial aspect of the psoas muscle (PM). The FN is held with forceps. S, superior; M, medial.

In the S-FICB approach, a difference was observed in the spreading patterns and involved nerves depending on the volume of dye used. When using 60 mL of dye, more extended spreading patterns to lumbar plexus branches were observed (Table 1). When using 30 mL of dye, spread to the GFN, LFCN, and FN was observed at the lateral border of the psoas muscle, but the ON was not stained (Figure 2A). However, with 60 mL of dye, the dye consistently spread to all lumbar plexus branches (iliohypogastric nerve, ilioinguinal nerve, GFN, LFCN, FN, and ON) (Table 1 and Supplementary Figure S1). 

In the S-FICBs with 60 mL of dye, spreading was observed beneath the external iliac artery and vein, detaching the pelvic fascia from the arcuate line and spreading to the ON (Figure 2B). In the S-FICBs with 60 mL of dye, spreading to the ON was confirmed on the posteromedial aspect of the psoas muscle and around the pelvic inlet (S1 level) in the retroperitoneal space (Figure 2C). In one S-FICB with 60 mL of dye, spreading to the lumbosacral trunk was observed. In all S-FICBs, dye spreading was observed from the inguinal ligament to the upper femoral region; however, the dye was located only on the posterolateral side of the femoral sheath close to the fascia iliaca. Furthermore, the LFCN and FN were stained; however, at the level below the inguinal ligament, the ON was not stained regardless of the dye volume used.

### 3.2. Dye Spreading Patterns in the I-FICBs

**Figure 3:**
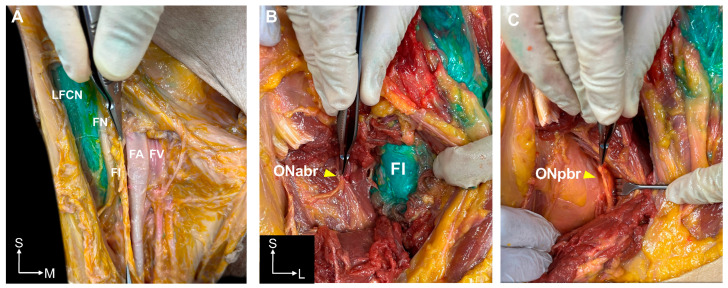
Comparison of dye spreading patterns in the infrainguinal fascia iliaca compartment block (I-FICB) using 30 mL and 60 mL of dye solution. (**A**). The lateral femoral cutaneous nerve (LFCN) and femoral nerve (FN) were stained in an I-FICB using 30 mL of dye. (**B**). The anterior branch of the obturator nerve (ON) was not stained in an I-FICB using 60 mL of dye. The fascia iliaca compartment contained the dye. (**C**). The posterior branch of the ON also was not stained in the I-FICB using 60 mL of dye. FI, fascia iliaca; FA, femoral artery; FV, femoral vein; ONabr, anterior branch of the obturator nerve; ONpbr, posterior branch of the obturator nerve; S, superior; M, medial; L, lateral.

In the I-FICBs, differences in spreading were not found in the involved nerves based on the volume of dye used (Table 1). Dye spreading in the cephalic to caudal direction was consistently observed, and both the LFCN and FN were stained with dye in all injections regardless of dye volume (Figure 3A). The dye was contained in the fascia iliaca compartment (a strong anatomical barrier) and spread into the medial compartment of the thigh was limited (Figure 3B). The dye did not spread to the anterior or posterior branch of the ON at the level of the fascia iliaca in any I-FICBs (Figure 3B,C). In I-FICBs with 60 mL of dye, there was spreading from the upper femoral region to the inguinal and abdominal region but not to any nerves (Supplementary Figure S2). In all I-FICBs, dye was found in the posterolateral side of the femoral sheath close to the fascia iliaca regardless of the volume used.

## 4. Discussion

The results of the present study confirmed a clear difference in the overall injectate spread pattern between S-FICB and I-FICB in cadavers. In addition, the volume of injectate differently affected the extent of spread between the two FICB approaches. Extended spreading of dye in a cranial direction to the branches of the lumbar plexus in a volume-dependent manner was observed in S-FICBs. Thus, most branches of the lumbar plexus, including the ON, were stained in S-FICBs using a high volume of dye (60 mL). However, in I-FICBs, ON staining was not observed even when a high volume of dye was used, and the predominant spreading pattern was in a caudad direction. Compared with I-FICB, S-FICB with 40 mL of injectate resulted in more consistent spreading in a cranial direction under the fascia iliaca and around the psoas muscle on MRI in living subjects [11]. In this context, S-FICB appears to have superior analgesic efficacy than I-FICB [12,13,14] because S-FICB could anatomically lead to injectate spreading more widely and closer to the lumbar plexus. 

In the present study, both S-FICB and I-FICB consistently showed LFCN and FN staining when more than 30 mL of injectate was used in cadavers. The ON staining was only observed in S-FICBs when 60 mL of dye was used. This result supports a previous cadaver study in which the minimum effective volume of dye required to stain the ON in S-FICB was 62.5 mL in 90% of cases [15]. However, the potential disruption of fascial structure integrity caused by a high-pressure injection of a high volume of injectate affected the extent of dye spread in a cadaver model [16]. In a recent study, the ON could not be anatomically involved in the S-FICB approach because both the fascia iliaca and pelvic fascia are attached to the pelvic brim [16,17]. Thus, a proposed mechanism [16] that indicates a physical communication between the fascia iliaca and the retroperitoneal space artificially created by S-FICB when using 60 mL of dye could not be excluded in the present study. Furthermore, from a clinical perspective, 60 mL of injectate is much higher than the conventionally used volume, raising concern about potential local anesthetic toxicity or prolonged quadriceps weakness in living subjects. Thus, further studies are needed to clarify whether using a high volume of local anesthetics in S-FICBs is effective and safe in clinical practice. 

In the current study, the ON was not stained in the I-FICB approach regardless of dye volume. The extrapelvic portion of the fascia iliaca is firmly connected to the hip joint capsule, spanning both sides of the iliopsoas tendon and muscle, along with the iliopectineal arch and inguinal ligament. This robust fascia iliaca barrier effectively separates the vascular and muscular compartments below the inguinal ligament and merges with the pectineus muscle tendon (iliopectineal fascia), creating a fused barrier tightly adhered to the hip joint capsule from the iliopectineal arch to the lesser trochanter [16,17]. Any injectate in the fascia iliaca compartment would need to traverse this robust barrier to spread to the ON that courses along the deep side of the pectineus muscle. Thus, the barrier effectively seals the fascia iliaca compartment, preventing the spread of injectate toward the ON in I-FICBs. The PEricapsular Nerve Group (PENG) block is a fascial plane block in which local anesthetic is injected in the fascial plane between the psoas tendon and the ilium, aiming to block the articular branches of the FN, ON, and accessory ON, which provide sensory innervation to the anterior hip joint capsule [18]. In our study, the dye did not spread deep into the iliopsoas muscle and onto the iliopubic eminence in the I-FICB approach. Indeed, compared with I-FICB, the PENG block provided superior analgesia effects and decreased opioid consumption in patients after surgery for hip fractures [19,20].

According to these results, the commonly administered volume of local anesthetics probably does not block the ON in S-FICBs and I-FICBs, hindering complete blockage of nerve innervation to the hip joint. In two randomized trials, reduction was not found in opioid consumption or pain intensity following I-FICB for hip arthroplasty compared with the sham block group [10,21]. The results of some meta-analysis studies also showed no significant beneficial effect on postoperative analgesia when using the I-FICB for hip surgery [22,23]. Furthermore, in a recently published study, S-FICB did not confer a significant opioid-sparing effect or reduction in pain score compared with sham block in patients who underwent hip surgery under spinal anesthesia [8,24]. The innervation of the hip joint capsule is highly variable, but its primary nerve supply seems to be from the nerve to quadratus femoris and ON [25]. Thus, although S-FICB was developed to obtain more consistent results than I-FICB, an additional ON block as a supplement to FICB, regardless of approach, is applied for postoperative analgesia in hip surgery.

Several limitations of the current study should be considered when interpreting the findings. Postmortem changes in the muscle and fascial structures can significantly affect the diffusion of the injectate. In addition, the results of this study cannot reflect delayed diffusion after FICBs that would occur with respiratory or abdominal movement in living subjects. One of the limitations of this study is the small sample size, with only three cadavers per group. Additionally, no statistical tests were employed, which limits the generalizability of the findings and the ability to draw definitive conclusions. Further studies with larger sample sizes and appropriate statistical analysis are needed to validate and expand upon these results. The ultra-high field diffusion tensor imaging technique could also be considered in further study to more accurately assess dye spread. 

## 5. Conclusions

The results of this cadaveric evaluation showed that the obturator nerve might not be affected in either suprainguinal- fascia iliaca compartment block or infrainguinal- fascia iliaca compartment block with the commonly used volume of injectate (30 mL) in clinical practice. The lateral femoral cutaneous nerve and femoral nerve were consistently stained in all injections. In addition, extended spreading to the lumbar plexus including the obturator nerve occurred in cadavers when a high volume of injectate (60 mL) was used in the suprainguinal- fascia iliaca compartment block but not in infrainguinal- fascia iliaca compartment block.

## Figures and Tables

**Table 1:** Comparison of the stained lumbar plexus branches based on dye solution volume (30 mL or 60 mL) between the S-FICB and I-FICB approaches.

Nerves	S-FICB		I-FICB	
	30 mL (*n* = 3)	60 mL (*n* = 3)	30 mL (*n* = 3)	60 mL (*n* = 3)
Ilioinguinal nerve	---	+++	---	---
Iliohypogastric nerve	---	+++	---	---
Genitofemoral nerve	+++	+++	---	---
Lateral femoral cutaneous nerve	+++	+++	+++	+++
Femoral nerve	+++	+++	+++	+++
Obturator nerve	---	+++	---	---

- indicates not stained, + indicates stained; FICB, fascia iliaca compartment block; S-FICB, suprainguinal fascia iliaca compartment block; I-FICB, infrainguinal fascia iliaca compartment block.

## Data Availability

Research data will be available upon request to the corresponding author.

## Supplementary Materials

The following supporting information can be downloaded at: https://www.mdpi.com/article/10.3390/medicina60091391/s1, Figure S1: The nerve involvement of the suprainguinal fascia iliaca compartment block using 60 mL of dye (The psoas muscle was removed); Figure S2: A horizontal section of the pelvic region after the infrainguinal fascia iliaca compartment block using 30 mL of dye.
