# Supplementary material for: Comparison of Dye Spread Pattern and Nerve Involvement between Suprainguinal and Infrainguinal Fascia Iliaca Blocks with Different Injectate Volumes: A Cadaveric Evaluation
Source: Medicina (Kaunas). 2024 Aug 25;60(9):1391. doi: 10.3390/medicina60091391 (PMC11433574; doi:10.3390/medicina60091391)
Supplement: Supplementary file 1 [file medicina-60-01391-s001.zip › Supplementary Figure S1.pdf]

Supplemental Figure S1. The nerve involvement of the suprainguinal fascia iliaca compartment block using 60ml of dye (The psoas muscle was removed)

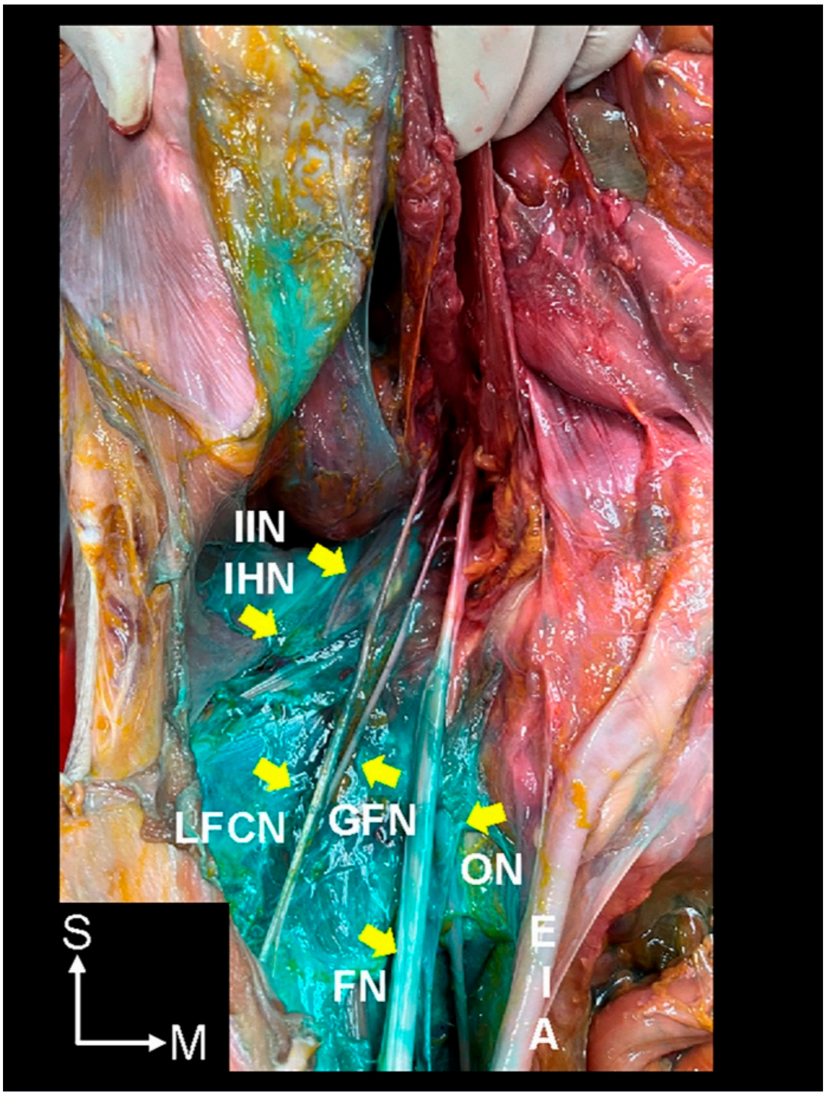

The dye consistently spread to all lumbar plexus branches (iliohypogastric nerve (IHN), ilioinguinal nerve (IIN), genitofemoral nerve (GFN), lateral femoral cutaneous nerve (LFCN), femoral nerve (FN), and obturator nerve (ON)). All yellow arrows indicate nerves. EIA, external iliac artery; S, superior; M, medial.
