# Supplementary material for: Comparison of Dye Spread Pattern and Nerve Involvement between Suprainguinal and Infrainguinal Fascia Iliaca Blocks with Different Injectate Volumes: A Cadaveric Evaluation
Source: Medicina (Kaunas). 2024 Aug 25;60(9):1391. doi: 10.3390/medicina60091391 (PMC11433574; doi:10.3390/medicina60091391)
Supplement: Supplementary file 1 [file medicina-60-01391-s001.zip › Supplementary Figure S2.pdf]

Supplementary Figure S2. A horizontal section of the pelvic region after the infrainguinal fascia iliaca compartment block using 30ml of dye

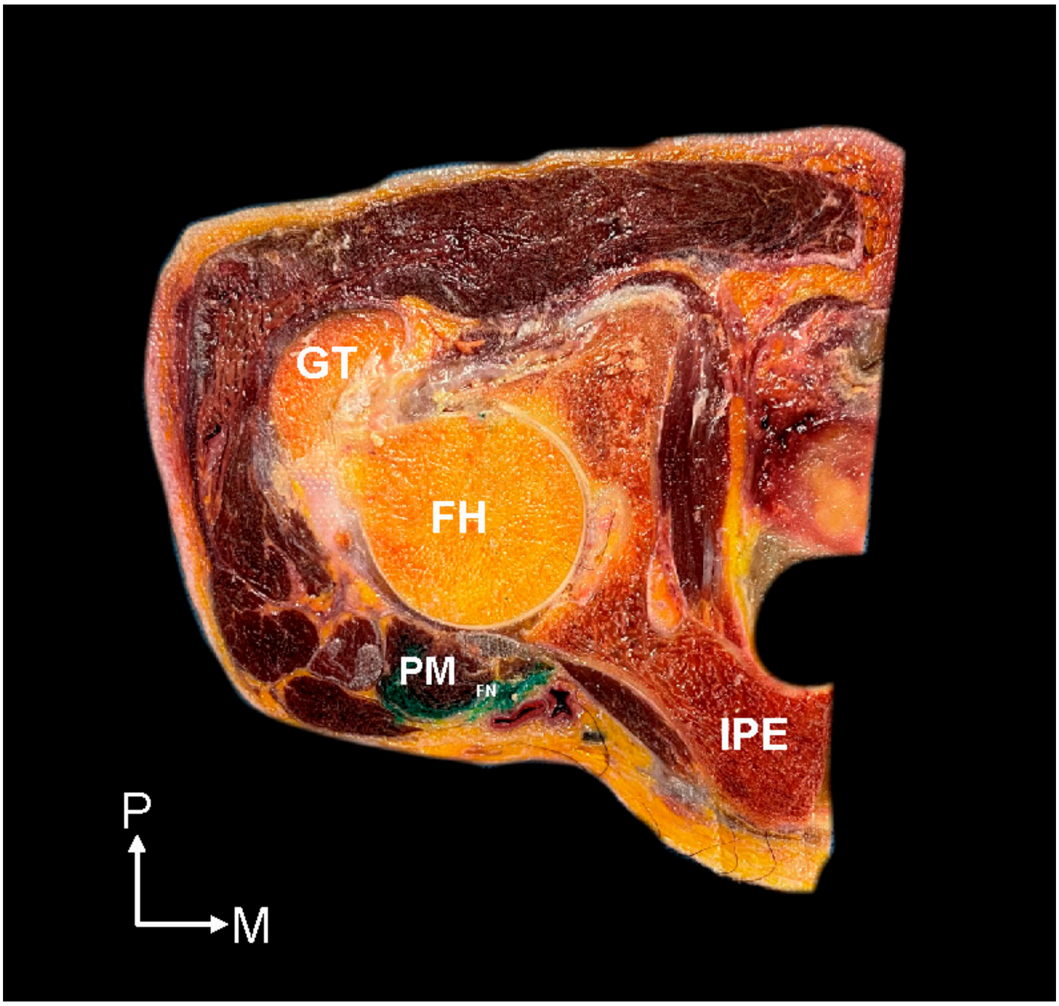

In the infrainguinal fascia iliaca compartment block using 30mL of dye, the dye did not spread deep into the iliopsoas muscle and onto the iliopubic eminence. GT, greater trochanter; FH, femoral head; PM, psoas muscle; FN, femoral nerve; IPE, iliopubic eminence; P, posterior; M, medial.
